# Outcomes of immediate dental implants in vascularised bone flaps for mandibular reconstruction

**DOI:** 10.1111/ans.18427

**Published:** 2023-04-07

**Authors:** Vinay Tumuluri, David Leinkram, Catriona Froggatt, Masako Dunn, James Wykes, Jasvir Singh, Tsu‐Hui (Hubert) Low, Carsten E. Palme, Dale Howes, Jonathan R. Clark

**Affiliations:** ^1^ Faculty of Health and Medical Sciences, School of Dentistry University of Adelaide Adelaide Australia; ^2^ Department of Head and Neck Surgery Sydney Head and Neck Cancer Institute, Chris O'Brien Lifehouse Sydney New South Wales Australia; ^3^ Sydney Medical School, Faculty of Medicine and Health The University of Sydney Sydney New South Wales Australia; ^4^ Royal Prince Alfred Institute of Academic Surgery Sydney Local Health District Sydney New South Wales Australia; ^5^ Department of Otolaryngology – Head & Neck Surgery, Faculty of Medicine and Health Sciences Macquarie University Sydney New South Wales Australia; ^6^ Sydney Dental School, Faculty of Medicine and Health The University of Sydney Sydney Australia

**Keywords:** dental implants, head and neck cancer, immediate implants, oral and maxillofacial surgery, surgical reconstruction

## Abstract

**Background:**

The aim of this study is to assess the outcomes of immediate implant placement for dental rehabilitation following mandibular reconstruction with vascularised bone flaps in a single Australian tertiary cancer centre.

**Methods:**

A retrospective analysis of patients who underwent immediate dental implant or delayed placement in vascularised bone flaps was performed. Primary outcome measures assessed included the number of implants placed, operative time, complication rates, time to radiotherapy initiation, dental rehabilitation rates and time to dental rehabilitation.

**Results:**

In total, 187 dental implants were placed in 52 patients, of which 34 patients underwent immediate implant placement and 18 had delayed implant placement. There were no significant differences in the postoperative complication rate (32% immediate vs. 33% delayed, *P* = 0.89) or time to postoperative radiotherapy (median 42 days immediate vs. 47 days delayed, *P* = 0.24). Dental rehabilitation was achieved in 62% of the immediate cohort versus 78% of the delayed cohort. The time to be fitted with a dental prosthesis was significantly shorter in the immediate cohort (median 150 days immediate vs. 843 days delayed, *P* = 0.002).

**Conclusions:**

The placement of immediate dental implants at the time of primary reconstruction of the mandible is a safe procedure and facilitates timely dental rehabilitation.

## Abstract 2

The aim of this study is to assess the safety outcomes of immediate implant placement for dental rehabilitation following mandibular reconstruction using vascularised bone flaps. A retrospective analysis of patients who underwent immediate or delayed dental implants in vascularised bone flaps was performed. Our results suggest the placement of immediate dental implants within vascularized bone flaps is a safe procedure that does not prolong the time to adjuvant radiotherapy.

## Introduction

Segmental mandibulectomy is a common treatment for locally aggressive tumours involving the mandible, of which oral cavity squamous cell carcinomas (OSCC) and ameloblastomas are the most prevalent indication.^1^ Reconstruction with vascularised free bone flaps post mandibular resection has substantially improved the functional and aesthetic outcomes of patients requiring these radical procedures and provided robust tissue for post‐operative radiotherapy when indicated.

The objective of osseous reconstruction has evolved from restoration of the mandibular continuity to functional rehabilitation of the oral cavity.^2^, ^3^, ^4^ This requires the careful placement of bone to facilitate the optimal positioning of dental implants as determined by the planned dental prosthesis. Occlusal‐based reconstructions using virtual surgical planning (VSP) have been associated with improved quality of life outcomes.^5^, ^6^ VSP has enabled the reconstructive surgeon to work closely with a prosthodontist to guide the virtual position of the reconstructed mandible. Custom (patient specific) surgical guides and titanium fixation plates are then manufactured ahead of surgery to facilitate the placement of the bone segments according to the digital plan.^7^

Historically, patients were assessed for dental rehabilitation after recovery from surgery and adjuvant therapy if required. Dental implants were placed several months after the initial surgery, followed by exposure of the implants after osseointegration, vestibuloplasty, and finally restoration with an implant supported prosthesis. Adell *et al*. found that only 50% of patients were dentally rehabilitated and the average time from resection to being fitted with a dental prosthesis was 2 years.^8^ It is likely that the rate of dental rehabilitation in Australian cancer centres is lower again due to the high cost of dental rehabilitation and lack of dedicated infrastructure.

With the development of VSP, several centres have moved towards primary implant placement at the time of the mandibulectomy with the aim of increasing the proportion of patients who are dentally rehabilitated and decreasing the time taken to achieve this.^9^, ^10^, ^11^, ^12^, ^13^, ^14^, ^15^, ^16^, ^17^ Longer operative times and increased surgical procedures associated with primary implant placement could increase treatment morbidity, which may impact disease control if radiotherapy is delayed.^18^ Allen *et al*., demonstrated no increase in surgical complications in a cohort of 61 patients who underwent mandibular resections for malignant disease.^19^ Additionally, there was no significant increase in the time to commencement of adjuvant radiotherapy.

Over recent years, the practice at our institution has also evolved to incorporate the immediate placement of dental implants at the time of free flap surgery when possible. Our hypothesis is that patients undergoing immediate implant placement will have a shorter time to dental rehabilitation, but this may come at the expense of a higher complication rate, which could in turn delay the commencement of adjuvant therapy. The primary aim of this study is to compare the outcomes of patients undergoing VSP‐guided bone flap reconstruction of the mandible and primary dental implant placement with delayed implant placement in terms of complication rates, operative time, time to initiation of radiotherapy, time to being fitted with a dental prosthesis and implant outcomes.

## Methods

Chris O'Brien Lifehouse (COBLH), Sydney, Australia prospectively maintains a jaw reconstruction database which collects clinical, pathological, quality of life, functional outcomes, and costing data on all patients who have undergone mandibulectomy or maxillectomy (HREC/16/RPAH/510). The database was constructed in 2020 and contains retrospective data on 267 patients treated at Royal Prince Alfred Hospital or COBLH from 13 February 2011 to 6 December 2020 and prospective data on 85 patients from 16 December 2020 to 19 January 2022.

This is a retrospective analysis of all patients who underwent mandible resection from 2011 to 2021 and had vascularised free bone flaps using occlusal‐based VSP and dental implant placement. Patients were excluded where complete data could not be obtained.

The primary outcomes of interest were dental rehabilitation rate and time to dental prosthesis placement. Secondary outcomes of interest were time to radiotherapy, length of stay, and complications. Patients were categorized into immediate dental implant placement (IIP) or delayed implant placement (DIP) groups. Study variables include sex, age, smoking, diabetes history, primary tumour type and its anatomical location, number of bone segments used, the number of implants placed and dental rehabilitation outcomes, time to radiotherapy commencement, length of hospital stay for the reconstruction and the readmission rate as well as all in‐patient complications. Complications were categorized according to the Clavien‐Dindo classification.^20^

### Patient selection and rehabilitation process

As mentioned, there has been an evolution of the timing for implant placement. At present, the option of dental rehabilitation is discussed with all patients undergoing mandibular reconstruction in consultation with the prosthodontic team. Patients are advised of the associated costs and requirements for maintenance of implant hygiene, potential complications, and additional procedures required. After informed consent, all patients are given the option to proceed with immediate implant placement if there is sufficient time to allow for planning. The protocol for implant placement and dental restoration was the same for the delayed and immediate implant groups. Following implant placement, multiunit abutments are secured to the implants and an impression is taken of the implant location using impression copings. Healing caps are placed onto the multi‐unit abutments and the implant/abutment complexes are buried usually beneath the skin paddle of the free flap. Prior to implant exposure, a stent/interim dental prosthesis is 3D printed. The implants are then exposed, and the interim bridge/stent is luted to cylinders (Southern^R^ TMC) using Luxatemp^R^ resin. The bridge is removed typically 2 months following placement, and the multi‐unit abutments are optically scanned to allow precise registration of implant locations. A new provisional bridge is constructed, or a definitive prosthesis is fabricated and fitted. The timing to definitive prosthesis depends on tissue healing, interim prosthesis condition, and patient preference which is often influenced by financial considerations given that the patients are usually charged external laboratory fees.

### Statistical analysis

Data are maintained securely in a Sydney Local Health District REDCap database. Patient demographics, presenting pathologies and surgical outcomes were exported for analysis in Stata version 12.0 SE (StataCorp LP). Univariable categorical data were analysed using the chi‐square test a two‐sample *t*‐test used for normally distributed continuous variables and the Mann–Whitney *U*‐test for non‐parametric data. Time to radiotherapy commencement was analysed as both continuous and categorical variables with delay in commencement of radiotherapy defined as greater than 6 weeks from surgery. Multivariable analysis was performed using logistic regression for binary outcomes (complications), reported as odds ratios (OR) with 95% confidence intervals (95% CI), and linear regression was used for continuous outcomes (time to dental rehabilitation) after normalization of errors (square root transformation) adjusting for the effect of statistically and clinically significant covariates. A two‐sided *P*‐value <0.05 was considered significant.

## Results

### Patient demographics and treatment findings

A total of 52 patients who had dental implants placed during or after vascularised bone flap reconstruction of the mandible were included (Table 1). There were 34 patients (65%) who underwent IIP at the time of free flap surgery and 18 patients (35%) had DIP at a later stage. The median follow‐up in the immediate implant cohort was 36 days (range; 2–1159 days) and in the delayed implant cohort was 581 days (range; 4–3435 days).

**Table 1:** Patient demographics and clinical findings

	Immediate (*n* = 34)	Historical (*n* = 18)	*P*
Sex			0.580
Male	20 (59%)	6 (33.3%)	
Female	14 (41%)	12 (66.6%)	
Mean age (years)	58.4	57.6	0.688
Indication			
Malignancy	28 (82.4%)	11 (61.1%)	
Osteoradionecrosis	3 (8.8%)	4 (22.2%)	
Defect following previous surgery	3 (8.8%)	3 (17%)	
Primary tumour type			0.060
Squamous cell carcinoma	19 (67.9%)	5 (45.5%)	
Ameloblastoma	4 (14.3%)	1 (9.1%)	
Osteosarcoma	3 (10.7%)	2 (18.2%)	
Adenoid cystic carcinoma	1 (3.6%)	0 (0%)	
Spindle cell carcinoma	1 (3.6%)	0 (0%)	
Basal cell carcinoma	0 (0%)	1 (9.1%)	
Mucoepidermoid carcinoma	0 (0%)	2 (18.2%)	
T classification			0.579
T1	0 (0%)	1 (9.1%)	
T2	2 (7.1%)	0 (0%)	
T3	1 (3.6%)	0 (0%)	
T4	18 (64.3%)	7 (63.6%)	
N/A	7 (25%)	3 (27.3%)	
History of prior head and neck malignancy			0.495
Yes	5 (14.7%)	4 (22.2%)	
No	29 (85.3%)	14 (77.8%)	
Smoking			0.006
Yes	5 (14.7%)	9 (50.0%)	
No	29 (85.3%)	9 (50.0%)	
Diabetes			0.674
Yes	3 (8.8%)	1 (5.5%)	
No	31 (91.2%)	17 (94.5%)	
Bone flap used for reconstruction			0.896
Fibula	29 (85.3%)	14 (77.8%)	
Deep circumflex iliac artery	4 (11.8%)	2 (11.1%)	
Scapula	1 (2.9%)	2 (11.1%)	
Bone flap laterality			0.111
Right	16 (47.1%)	5 (27.8%)	
Left	18 (52.9%)	13 (72.2%)	
Number of bone segments used			0.527
1	10 (29.4%)	7 (38.9%)	
2	16 (47.1%)	8 (44.4%)	
3	8 (23.5%)	2 (11.1%)	
4	0 (0%)	1 (5.5%)	

A summary of patient demographics and clinical findings are presented in Table 1. The median age was 60.7 years (range: 23–83 years). There was an even sex distribution overall (M:F 26:26), however a greater proportion of the DIP cohort were female (*n* = 12, 67%) compared with the IIP cohort (*n* = 14, 41%) (*P* = 0.58) and a greater proportion of the DIP cohort were smokers (*n* = 9, 50%) compared with the IIP cohort (*n* = 5, 15%) (*P* = 0.006).

The majority of patients had immediate osseous reconstruction following tumour ablation, however this was more frequent in the IIP cohort (*n* = 28, 82%) versus the DIP cohort (*n* = 11, 61%) (*P* = 0.18).

Other indications for bony‐free flap reconstructions were osteoradionecrosis and delayed (secondary) reconstruction following primary surgery. OSCC was the most common tumour diagnosis across both cohorts (*n* = 24, 46%), however, this was more common in the IIP cohort (*n* = 19, 68%) compared to the DIP (*n* = 5, 46%) (*P* = 0.06). The fibula free flap was the most common reconstruction used (*n* = 43, 83%), with the remainder either undergoing a scapula (*n* = 3, 6%) or a deep circumflex iliac artery (DCIA; *n* = 6, 12%) free flap. There were 17 patients (33%) who underwent adjuvant radiotherapy with the majority of these undergoing IIP (*n* = 13, 76%). There were no other statistically significant differences between the two groups.

### Implant outcomes

In total 187 dental implants were placed across 52 patients. Of these, 123 implants were placed immediately across 34 patients (median number of implants = 3, range 2–8) (Table 2), compared with 64 implants placed across 18 patients in the DIP cohort (median number of implants = 4, range 2–7) (Table 4). The median time from osseous free flap reconstruction to implant placement in DIP cohort was 398 days (range 101–2472 days). There were three (2%) implants removed due to lack of osseointegration, of which two (3%) were within the DIP cohort and one (1%) was in the IIP cohort (*P* = 0.23).

**Table 2:** Adjuvant radiotherapy details

	Immediate (*n* = 34)	Delayed (*n* = 18)	*P*
	34 (65%)	18 (35%)	
Radiation			0.242
Adjuvant	13 (38.2%)	4 (22.2%)	
None	21 (61.8%)	14 (77.8%)	
Mean time to Adjuvant XRT initiation (SD)	43.9 days (12.9)	47.6 days (16)	0.028
Median time to Adjuvant XRT completion (range)	42 days (28–62)	47 days (32–64)	

### Operative time, complications, and time to postoperative radiotherapy

The mean operative time for osseous free flap reconstruction was longer in the IIP group at 555 min (9 h, 15 min) compared with 487 min (8 h, 7 min) in the DIP cohort (*P* = 0.03) (Table 3).

**Table 3:** 90‐day postoperative complications

	Immediate (*n* = 34)	Delayed (*n* = 18)	*P*
	34 (65%)	18 (35%)	
Complication Grade			0.888
Grade I	6 (18%)	1 (6%)	
Grade IIIb	6 (18%)	5 (28%)	
Total	12 (35%)	6 (33%)	
Details			0.279
Fever/sepsis	2	2	
Osteoradionecrosis	0	1	
Ocular complications (eye pain/epithelial defect)	0	2	
Flap failure	2	0	
Neck wound complications/Iliac crest avulsion fracture	3	0	
Donor site wound complication	2	1	
Pseudomonas pneumonia	1	0	
Free flap ischemia	2	0	

There were 12 IIP patients (32%) who experienced complications compared to six patients (33%) in the DIP cohort. There were six patients in the IIP cohort experienced Clavien‐Dino Grade I Clavien‐Dindo and six Grade IIIb complications. There were five patients in the DIP cohort experienced Grade I complications and one patient had a Grade IIIb complication. This included one free flap failure and three patients who returned to theatre in the IIP for wound dehiscence, flap ischaemia, and neck wound infection. There was no significant difference in overall complication rates between IIP and DIP cohorts (*P* = 0.89). After adjusting for the effect of smoking, diabetes, age, and preoperative radiotherapy there was no significant difference in the odds of developing a complication between IIP and DIP cohorts (OR 0.90, 95% CI 0.23–3.40, *P* = 0.87).

The mean time to adjuvant radiotherapy in IIP cohort was 44 days (range 28–62 days) postoperative compared to 48 days (range 32–64 days) in the DIP cohort (*P* = 0.03). There were three patients (23%) in the IIP cohort experienced delays to radiotherapy commencement, defined as greater than 6 weeks from surgery, compared with one patient (25%) in the DIP cohort.

### Dental prosthetic outcomes

At the end of the study period, 21 patients (62%) within the IIP cohort and 13 patients (72%) in the DIP cohort completed dental rehabilitation (*P* = 0.24) (Table 4). In addition, a further eight patients (24%) from the IIP cohort had dental rehabilitation in progress during the study period. The median time to being fitted with a dental prosthesis in the DIP cohort was 843 days compared to 150 days in the IIP cohort (*P* = 0.002). After adjusting for the effect of smoking (*P* = 0.04), age (*P* = 0.01), and a diagnosis of cancer (*P* = 0.005), there remained a statistically significant increase in time to dental rehabilitation between IIP and DIP cohorts (*P* < 0.001).

**Table 4:** Outcomes of patients with dental implants

	Immediate (*n* = 34)	Historic cohort (*n* = 18)
	34 (65%)	18 (35%)
Total Implants	123	64
Mean implants per patient	3.62	3.55
Vestibuloplasty		
Yes	15 (44.1%)	10 (55.6%)
No	19 (55.9%)	8 (44.4%)
Median time to Vestibuloplasty (days)	181	638
Dental rehabilitation completed, No (%)	21 (61.8%)	13 (72.2%)

## Discussion

This study of 52 patients undergoing dental implant placement in osseous free flap reconstructions of the mandible demonstrates that the complication rates between IIP and DIP groups is similar, with similar time to postoperative radiotherapy. A high proportion of patients receiving DIP were dentally rehabilitated, however, the time to dental rehabilitation was much shorter in the IIP cohort. Whilst strong conclusions are difficult due to heterogeneity and limited sample size, it appears that IIP is safe from an oncological perspective and achieves more rapid dental rehabilitation.

Placement of a dental prosthesis improves quality of life and functional outcomes following ablation of the mandible^21^, ^22^ and therefore, the time taken to achieve this goal is important. On average, IIP reduced the time to dental prosthesis insertion from several years to under 6 months. The Alberta Reconstruction Technique (ART) is one of the first reported occlusion‐driven jaw reconstruction techniques with digitally planned dental implant placement.^10^ Seikaly *et al*. reported the outcomes of 15 patients who had immediate implants placed at the time of resection and had completed dental rehabilitation using the ART technique. The median time from reconstruction to dental rehabilitation was 27 months in the delayed implant cohort which is far longer than our IIP cohort (5 months). The ART protocol necessitates 6 months of healing post‐completion of cancer treatment and hyperbaric oxygen treatment was provided for patients who underwent radiotherapy. Further, at our institution, a dental prosthesis was inserted at the time of resection on several patients which greatly reduced the average time to dental restoration.^23^

The benefits of early dental rehabilitation are achievable for both the long‐term survivors of oral cancer and those who succumb to their disease. Both groups are prevalent in Australia where 5 year survival rates of OSCC are 66.1%.^24^

Patients within our IIP cohort underwent adjuvant radiotherapy in a mean time of 44 days compared with 48 days in the DIP cohort (*P* = 0.03). Resistance to the placement of immediate implants in the oncological patient, for fears of delay to commencement of radiotherapy was not supported by this study.

There were 12 patients with immediate dental implants placed who experienced post‐operative complications, of which three were major compared with six in the delayed cohort, (no major complications) within the 90‐day period following reconstructive surgery. Whilst the difference between the groups were not statistically significant, surgeons performing these procedures need to be aware for the potential of IIP to lengthen operating time and increase adverse outcomes. Placement of dental implants requires additional stripping of periosteum and manipulation of the osseous flap which can potentially lead to flap compromise. Abutments are placed into the implants and can become partially exposed, leading to wound infection. Further, apart from implant placement, dental impressions are also obtained which requires additional time and may compromise sterility. Irawati *et al*. found an 11% increase in post‐operative complications per hour of increased operative time in patients undergoing microvascular free flap surgery for reconstruction of head and neck defects.^18^ Hence, the increased hour of operating time observed in this study may be of great significance to patients, surgeons, and hospital resources.

Finally, the surgery is complex and often in a patient population with significant comorbidities. The additional technical component and time in theatre may contribute to serious adverse surgical outcomes. This needs to be balanced against the capacity for earlier fitting of a dental prosthesis, and the associated functional and quality of life advantages.

### Limitations

Questions not addressed by our study include whether or not primary implant placement delayed the time from diagnosis to treatment. Without a streamlined workflow, the planning of these cases can delay the primary resection which in turn may compromise the cancer treatment. The limited sample size, retrospective analysis, and highly heterogenous cohort make strong conclusions impossible. Furthermore, it is unlikely that the results demonstrated here are transferrable to other institutions performing lower volumes of complex cancer resections and reconstructions. The financial and insurance status of patients plays a large role in their dental rehabilitation and time to restoration. There is a need for further analysis to examine the impact that these factors have on patient's dental rehabilitation and integration into society. Further prospective multicentre studies are required to evaluate all of these factors across a variety of institutional practices.

## Conclusion

Placement of dental implants into vascularised bone flaps at the time of primary resection and reconstruction is an evolving paradigm. Multi‐disciplinary assessment, VSP and reconstructive techniques contributed to faster dental rehabilitation. The placement of immediate dental implants within vascularized bone flaps is a safe procedure that does not prolong the time to adjuvant radiotherapy.

## Conflict of interest

None declared.

## Author contributions

**Vinay Tumuluri:** Conceptualization; data curation; formal analysis; investigation; methodology; project administration; writing – original draft. **David Leinkram:** Supervision; writing – review and editing. **Catriona Froggatt:** Data curation; investigation; methodology; project administration; writing – review and editing. **Masako Dunn:** Data curation; investigation; methodology; project administration; writing – review and editing. **James Wykes:** Data curation; writing – review and editing. **Jasvir Singh:** Data curation; writing – review and editing. **Tsu‐Hui (Hubert) Low:** Data curation; writing – review and editing. **Carsten E. Palme:** Data curation; writing – review and editing. **Dale Howes:** Data curation; writing – review and editing. **Jonathan R. Clark:** Conceptualization; data curation; formal analysis; investigation; methodology; project administration; supervision; writing – review and editing.
